# Importance of Val567 on heme environment and substrate recognition of neuronal nitric oxide synthase

**DOI:** 10.1002/2211-5463.12503

**Published:** 2018-08-20

**Authors:** Inger K. Olsbu, Giorgio Zoppellaro, K. Kristoffer Andersson, Jean‐Luc Boucher, Hans‐Petter Hersleth

**Affiliations:** ^1^ Department of Biosciences Section for Biochemistry and Molecular Biology University of Oslo Norway; ^2^ Regional Centre of Advanced Technologies and Materials Department of Physical Chemistry Faculty of Science Palacky University in Olomouc Czech Republic; ^3^ UMR 8601CNRS‐University Paris Descartes France; ^4^ Department of Chemistry Section for Chemical Life Sciences University of Oslo Norway

**Keywords:** active‐site mutations, heme, nitric oxide, nitric oxide synthase, substrate analogues binding, UV‐visible spectroscopy

## Abstract

Nitric oxide (NO) produced by mammalian nitric oxide synthases (mNOSs) is an important mediator in a variety of physiological functions. Crystal structures of mNOSs have shown strong conservation of the active‐site residue Val567 (numbering for rat neuronal NOS, nNOS). NOS‐like proteins have been identified in several bacterial pathogens, and these display striking sequence identity to the oxygenase domain of mNOS (NOSoxy), with the exception of a Val to Ile mutation at the active site. Preliminary studies have highlighted the importance of this Val residue in NO‐binding, substrate recognition, and oxidation in mNOSs. To further elucidate the role of this valine in substrate and substrate analogue recognition, we generated five Val567 mutants of the oxygenase domain of the neuronal NOS (nNOSoxy) and used UV‐visible and EPR spectroscopy to investigate the effects of these mutations on the heme distal environment, the stability of the heme‐Fe^II^‐CO complexes, and the binding of a series of substrate analogues. Our results are consistent with Val567 playing an important role in preserving the integrity of the active site for substrate binding, stability of heme‐bound gaseous ligands, and potential NO production.

**Abbreviations**
BH_4_: (6R)‐5,6,7,8 tetrahydro‐l‐biopterin
DTT: dithiothreitol
ImH: imidazole
l‐Arg: l‐arginine
mNOS: mammalian NOS
nNOS: neuronal NOS
NOHA: N^ω^‐hydroxy‐l‐arginine
NOS: nitric oxide synthase
NOSoxy: oxygenase domain of NOS
PMSF: phenylmethylsulfonyl fluoride
WT: wild‐type

Nitric oxide (NO) is an important mammalian signaling molecule synthesized by nitric oxide synthase (NOS). There are three distinct isoforms of mammalian NOS (mNOSs), the neuronal, inducible, and endothelial NOS (n‐, i‐ and e‐NOS, respectively), that can be distinguished by their initial cellular identification, primary sequence, and modes of regulation 1, 2, 3. The structure of the three mNOS isoforms can be divided into two main parts: a N‐terminal oxygenase domain (NOSoxy) containing the heme prosthetic group, with binding sites for substrate l‐arginine (l‐Arg), and cofactor (6R)‐5,6,7,8‐tetrahydro‐l‐biopterin (BH_4_), and a C‐terminal reductase domain containing binding sites for cofactors, FMN, FAD, and NADPH. These two domains are connected by a calmodulin‐binding sequence 4, 5. NOS converts l‐Arg to l‐citrulline and NO in a two‐step oxidation with the intermediate formation of N^ώ^‐hydroxy‐l‐Arg (NOHA) in the first step (Fig. 1) 6.

**Figure 1:**
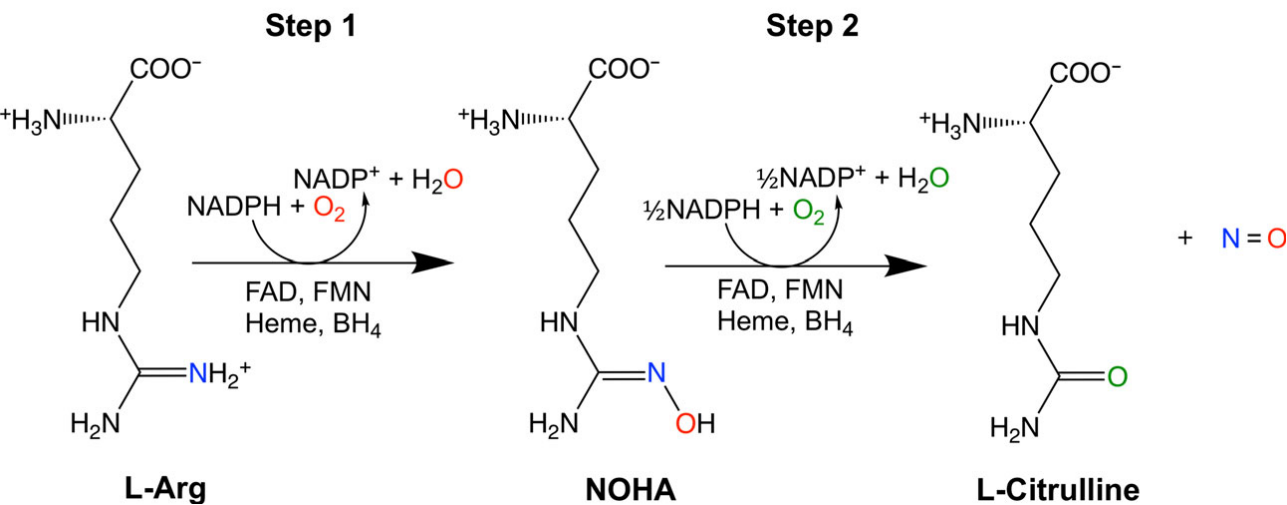
The two‐step reaction catalyzed by NOSs. The first step involves the initial hydroxylation of l‐Arg resulting in the formation of N^ω^‐hydroxy‐l‐arginine (NOHA) followed in a second step by the oxidation of NOHA to l‐citrulline and NO with overall consumption of 1.5 mol NADPH and 2 mol O_2_ per mol of l‐citrulline and NO formed.

Low levels of NO are important in a variety of physiological functions, such as neurotransmission, immune response, and vasodilation. High levels of NO are involved in several pathologies associated with oxidative stress phenomena such as atherosclerosis, Alzheimer's, Huntington's, and Parkinson's diseases, most of which are linked to the concomitant formation of superoxide, hydrogen peroxide, and peroxynitrite by a variety of enzymes including NOSs 7, 8, 9, 10, 11, 12, 13. These findings show important pharmacological challenges with respect to the elucidation of NOSs mechanism and the search and discovery of potent selective inhibitors 14, 15, 16

Despite the broad range of biological activity of the three mNOSs, the crystal structures of their oxygenase domains have shown a very strong conservation of the heme active‐site residues (Fig. 2A) 21, 22, 23, 24, 25 with identification of key amino acids involved in the binding of substrates l‐Arg and NOHA. Crucial hydrogen bonds are formed between the guanidine or hydroxyguanidino groups of l‐Arg and NOHA and the highly conserved Glu592 (rat nNOS numbering) thus governing their binding close to the heme (Fig. 2A) 22, 26, 27. Furthermore, the crystallographic studies showed a hydrogen‐bond network, involving l‐Arg, diatomic ligands, and an active‐site water molecule that could be involved in proton shuttling during catalysis 28, 29. It has been proposed that this water molecule is important in step 1 by hydrogen bonding to the high‐valent oxo intermediate, while step 2 propagates through a different mechanism 30, 31, 32. Previous studies have also identified hydrogen bonds between the OH‐group of the highly conserved Tyr588 and the α‐COOH‐group of l‐Arg and NOHA believed to be of importance in substrate recognition with mutations of Tyr588 strongly altering substrate binding and oxidation 33, 34. Although neither the substrate nor BH_4_ coordinate as heme iron ligands, it has been shown that their binding markedly influences the heme iron properties by shifting the heme iron spin equilibrium toward high‐spin state altering its electron properties and binding of ligands like CO and NO 35, 36, 37, 38, 39, 40, 41, 42.

**Figure 2:**
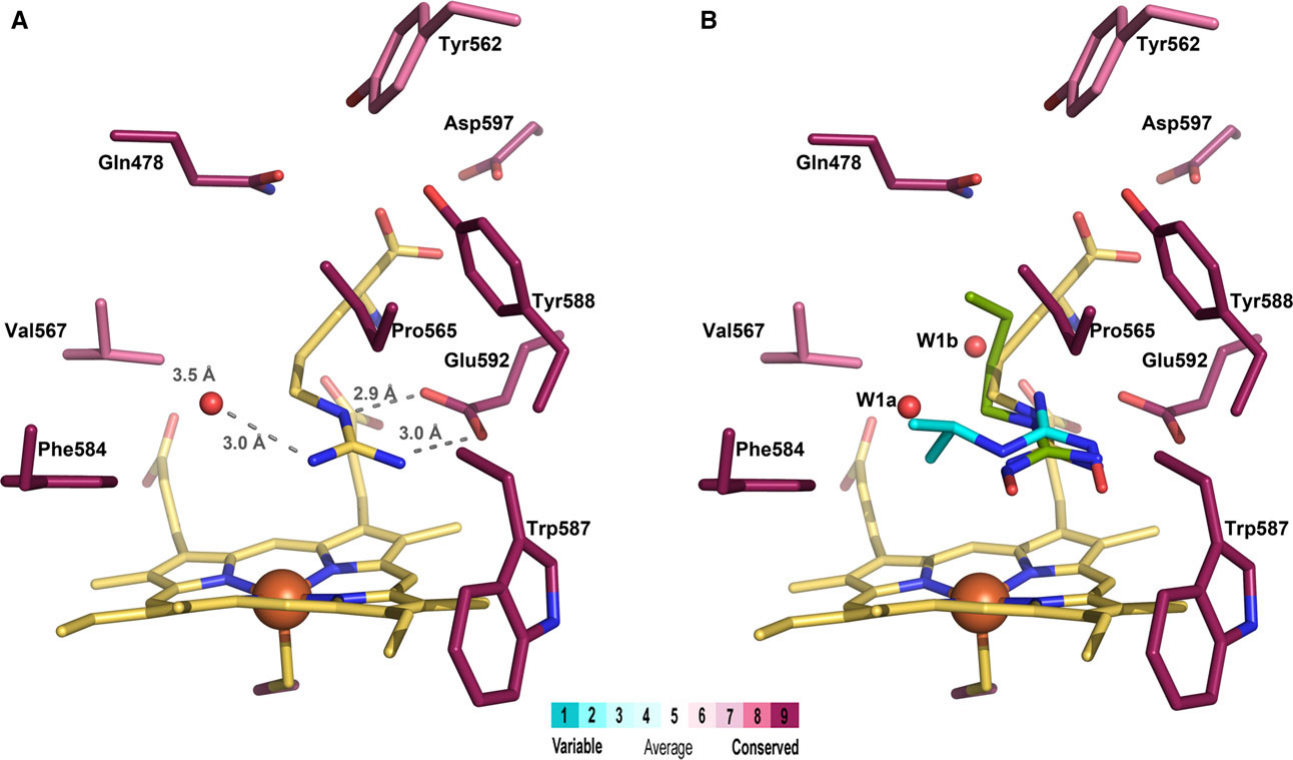
Structure of the nNOS active site (PDB id http://www.rcsb.org/pdb/search/structidSearch.do?structureId=1OM4) showing the main interactions between the substrate and the residues close to the heme. The residues are colored according to the conservation score determined by ConSurf 17, 18, 19, 20 from searching for homologous sequences of the rat nNOSoxy domain sequence (turquoise: least conserved, purple: most conserved). (A) Structure shown with l‐Arg and Water 1a (yellow, PDB id: http://www.rcsb.org/pdb/search/structidSearch.do?structureId=1OM4). (B) Same as A but in the presence of *N*‐butyl‐*N*’‐hydroxyguanidine (green, PDB id: http://www.rcsb.org/pdb/search/structidSearch.do?structureId=1M00) and *N*‐isopropyl‐*N*’‐hydroxyguanidine with structural water molecule (turquoise, PDB id http://www.rcsb.org/pdb/search/structidSearch.do?structureId=1LZZ) 24.

Mammalian NOSs have been shown to also catalyze the oxidation of non‐amino acids, *N*‐alkyl‐guanidines and *N*‐aryl‐*N*’‐hydroxy‐guanidines, with subsequent NO formation, following a similar oxidation as that of natural substrates l‐Arg and NOHA 43, 44, 45, 46, 47. Crystal structures of the NOSoxy domains have revealed a novel binding mode where the alkyl chains move toward a hydrophobic pocket at the heme distal site comprising highly conserved residues Phe, Pro, and Val (Fig. 2) 24. We have previously reported the importance of Val567 in full‐length nNOS for the binding and oxidation of l‐Arg, NOHA, and alternative substrates to NO 48. Furthermore, this Val residue is highly conserved among the different mNOSs and eukaryotic NOSs. However, in the majority of bacterial NOSs, this conserved Val has been replaced by an Ile believed to create a greater steric shielding of the heme prosthetic group. This results in a much slower NO dissociation compared to its mammalian counterpart 49, 50, 51.

In order to better understand the importance of this Val residue on the heme distal environment and substrate recognition, five nNOSoxy Val567 mutants were constructed. Here, we report the spectroscopic characterization of wild‐type (WT) nNOSoxy and five Val567 mutants and their ability to bind l‐Arg, NOHA, and a series of non‐amino acid guanidines.

## Materials and methods

### Chemicals

**Figure 3:**
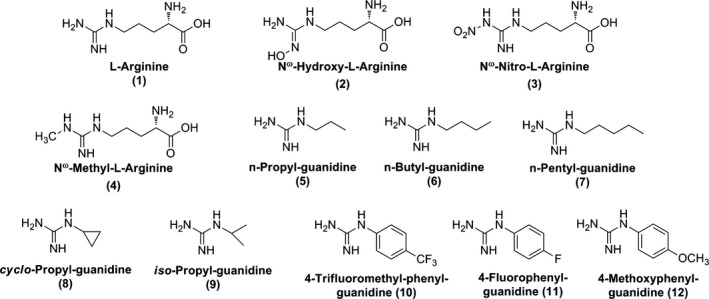
Structures of the studied guanidines **1–12**.

BH_4_ was purchased from Schircks Laboratories (Jona, Switzerland). l‐Arg **1**, N^ω^‐nitro‐l‐Arg **3**, N^ω^‐methyl‐l‐Arg **4,** and most of the other reagents were obtained from Sigma and were of the highest purity commercially available. The synthesis and physicochemical characteristics of NOHA **2**, n‐propyl‐guanidine **5**, n‐butyl‐guanidine **6**, n‐pentyl‐guanidine **7**,* cyclo*propyl‐guanidine **8**,* iso*propyl‐guanidine **9**, 4‐trifluoromethyl‐phenyl‐guanidine **10**, 4‐fluorophenyl‐guanidine **11,** and 4‐methoxyphenyl‐guanidine **12** have been previously described (Fig. 3) 32, 47.

### Protein expression and purification

The plasmid pCWnNOSoxy (aa 1–720 plus a 6‐histidine tag) 52 was transformed into BL 21 (DE3) cells (Novagen, Darmstadt, Germany). Cells were grown from freeze stocks in 100 mL Luria‐Bertani medium containing 100 μg·mL^−1^ ampicillin overnight at 37 °C with vigorous shaking. The overnight culture was diluted 100‐fold into 1 L Terrific broth. When cells had grown to an OD600 of 0.8–1, the cultures were cooled to 25 °C before induction by adding isopropyl β‐d‐1‐thiogalactopyranoside and δ‐aminolevulinic acid to final concentrations of 1 mm and 0.2 mm, respectively. Cultures were grown for 65–72 h at 20 °C before cells were harvested by centrifugation (~6400 g, 15 min). The cell paste was then stored at −20 °C prior to purification.

Cells were suspended in 200 mL buffer A [50 mm Tris/HCl pH 7.4, 250 mm NaCl, 20% glycerol, 1 mm DTT, 1 mm EDTA, 0.8 mg·mL^−1^ lysozyme, 1 mm phenylmethylsulfonyl fluoride (PMSF)] and a cocktail of protease inhibitors (pepstatin A, leupeptin, and antipain, 1 μg·mL^−1^ each) and lysed using sonication. Cell debris was removed by centrifugation (~48 000 g, 60 min at 4 °C). DNA was precipitated by adding streptomycin sulfate to a final concentration of 2.5% (w/v) and discarded after centrifugation at 48 000 g for 30 min at 4 °C. Finally, solid ammonium sulfate (0.291 g·mL^−1^) was added to the supernatant to precipitate proteins and the precipitate was removed by centrifugation (~15 000 g, 45 min at 4 °C). The precipitated proteins were dissolved in binding buffer B (50 mm Tris/HCl pH 7.4, 250 mm NaCl, 10% glycerol, 10 mm imidazole (ImH), 1 mm PMSF, and 1 mm DTT) and applied to a 5 ml HisTrap HP (GE Healthcare, Oslo, Norway) column pact with Ni Sepharose High Performance affinity resin using an ÄKTA purifier FPLC system (GE Healthcare). Proteins were eluted using a stepwise gradient of 10–50 mm ImH in purification buffer C (50 mm Tris/HCl pH 7.4, 250 mm NaCl, and 10% glycerol). The colored nNOSoxy fractions were pooled, and dialysis was performed to remove ImH using dialysis tubing of cellulose membrane in buffer D (50 mm Tris/HCl pH 7.4, 250 mm NaCl, 20% glycerol, 1 mm DTT). After 2 h at 4 °C, buffer was replenished and protein left to stir overnight at 4 °C. The protein was concentrated in a Centricon Plus‐70 filter unit (30‐kDa cutoff, Merck Millipore, Oslo, Norway). To avoid denaturation of nNOSoxy, all buffers contained 10 μm BH_4_. Purified WT nNOSoxy and Val567 mutants were more than 95% pure as determined by SDS/PAGE stained with Coomassie Blue 53, 54.

The heme concentration was measured using UV‐visible spectroscopy (see below), and samples were aliquoted, flash‐frozen in liquid nitrogen, and stored at −80 °C.

#### Mutagenesis

Site‐directed mutagenesis of the pCWnNOSoxy expression plasmid was performed using a QuikChange site‐directed mutagenesis kit (Stratagene, La Jolla, CA, USA). The mutations were incorporated into the primers as follows:

Val567Ser 5′ GGC‐CTC‐CCC‐GCT‐***AGC***‐TCC‐AAC‐ATG‐CTG‐CTG
Val567Thr 5′ GGC‐CTC‐CCC‐GCT‐***ACG***‐TCC‐AAC‐ATG‐CTG‐CTG
Val567Tyr 5′ GGC‐CTC‐CCC‐GCT‐***TAT***‐TCC‐AAC‐ATG‐CTG‐CTG
Val567Phe 5′ GGC‐CTC‐CCC‐GCT‐***TTT***‐TCC‐AAC‐ATG‐CTG‐CTG
Val567Arg 5′ GGC‐CTC‐CCC‐GCT‐***CGT***‐TCC‐AAC‐ATG‐CTG‐CTG

The mutations were confirmed by sequencing (GENOME express, Maylan, France) 500–800 consecutive/overlapping base pairs including the mutations sites.

### UV‐visible spectroscopy

Optical spectra were recorded using an Uvikon 941 spectrophotometer in 150‐μL quartz cuvettes. The concentration of nNOS was determined optically from the [CO‐reduced]‐[reduced] difference spectrum using Δε_444–470 nm_ = 76 mm ^−1^·cm^−1^ 55.

The stability of Fe^II^‐CO complexes of WT nNOSoxy and its mutants was monitored at room temperature (~25 °C) following the absorbance at 443 nm over time in the presence of 10 μm BH_4_ and in the absence or presence of 10 mm l‐Arg in 50 mm HEPES buffer pH 7.4. The changes in absorption at 443 nm were then plotted over time, and the percentage of remaining absorbance after 45 min were calculated.

The binding affinities of ImH, substrates l‐Arg and NOHA, and substrate analogues **3–12** for WT and Val567 mutants were determined by perturbation difference spectroscopy 37. The native enzymes (~1–2 μm) were incubated 5 min at 4 °C in 50 mm HEPES buffer pH 7.4 in the presence of 0.5 mm ImH and then equally divided into reference and sample cuvettes. After 2 min at room temperature, increasing concentrations of the studied compounds were added to the sample cuvette and equivalent amounts of buffer were added to the reference cuvette. Apparent dissociation constants (*K*_s,app_) were estimated by plotting the difference in absorbance between peak (~395 nm) and valley (~430 nm) as a function of added substrate or substrate analogue concentrations and fitting the data to a hyperbolic one‐site binding model by Origin (OriginLab Corp., Northampton, MA, USA). The *K*_s,app_ for substrates and substrate analogues does not take into account the dissociation constant of ImH. Effective *K*_s_ values can be deduced from the *K*_s,app_ and the *K*_s_ values for ImH, assuming simple competitive binding equilibrium 37.

### Electron paramagnetic resonance spectroscopy

The WT and mutants protein sample (~100 μm based on heme concentration, final volume 90 μL) were incubated at 4 °C in 50 mm HEPES buffer pH 7.4 containing 10% glycerol and 10 μm BH_4_ under argon. After 10 min, samples were flash‐frozen in quartz EPR tubes and stored at −80 °C prior to spectroscopic measurements. EPR spectra were recorded on a Bruker Elexsys 560 EPR spectrometer operating at X‐band frequency (9.66 GHz) equipped with an ER41116DM dual mode cavity using a He‐flow cryostat (ESR900 Oxford Instruments, Abingdon, UK) and an Oxford Instrument liquid helium probe. The following instrument settings were used: modulation frequency, 100 kHz; modulation amplitude, 0.75 mT; time constant, 0.02 s; scan width, 0.5 T; field sweep, 100 mT·min^−1^; center field, 255 mT; and sweep time 168 s, 4 mW applied microwave power, and averaged four scans.

### Homology modeling of mutations

To obtain an estimation of the probable orientation of the different Val567 mutants, they were subjected to homology modeling using SWISS‐MODEL 56, 57, 58, 59 with the mutated sequences as target sequences. The sequence similarity search through SWISS‐MODEL resulted in the structure with PDB id http://www.rcsb.org/pdb/search/structidSearch.do?structureId=3HSO to be the best fit, and this structure was utilized by Automodel for the homology modeling of the different mutants. The heme group was included (PDB id http://www.rcsb.org/pdb/search/structidSearch.do?structureId=3HSO) in the homology modeling. In a further step, l‐Arg was added to the five different models of the Val567 mutants, and a small geometry minimalization with Phenix was performed 60. Structure figures were generated with PyMOL (Schrödinger, LLC).

## Results

To investigate the importance of an active‐site residue at the hydrophobic site of the distal heme pocket of nNOSoxy, we mutated Val567 (nNOSoxy) into both different hydrophobic (Phe, Tyr) and polar (Arg, Ser, Thr) residues. Val to Phe and Tyr mutations would give information on steric hindrance and possible π–π interactions. Ser and Thr mutations were introduced to explore the effects of disrupting the hydrophobic pocket, and the effects of introducing a positive charge were investigated by introducing an Arg residue. Using established protocols, we successfully expressed WT nNOSoxy and five mutants from transformed *Escherichia coli*. WT nNOSoxy, and its Val567Ser, Val567Tyr, and Val567Thr mutants were purified in almost identical yield (3–5 mg·L^−1^ of culture), and Val567Phe was obtained in a slightly higher yield (6–8 mg·L^−1^ of culture), whereas Val567Arg was obtained in a lower yield (1–2 mg·L^−1^ of culture).

### UV‐visible spectroscopic properties of WT nNOSoxy and Val567 mutants

**Figure 4:**
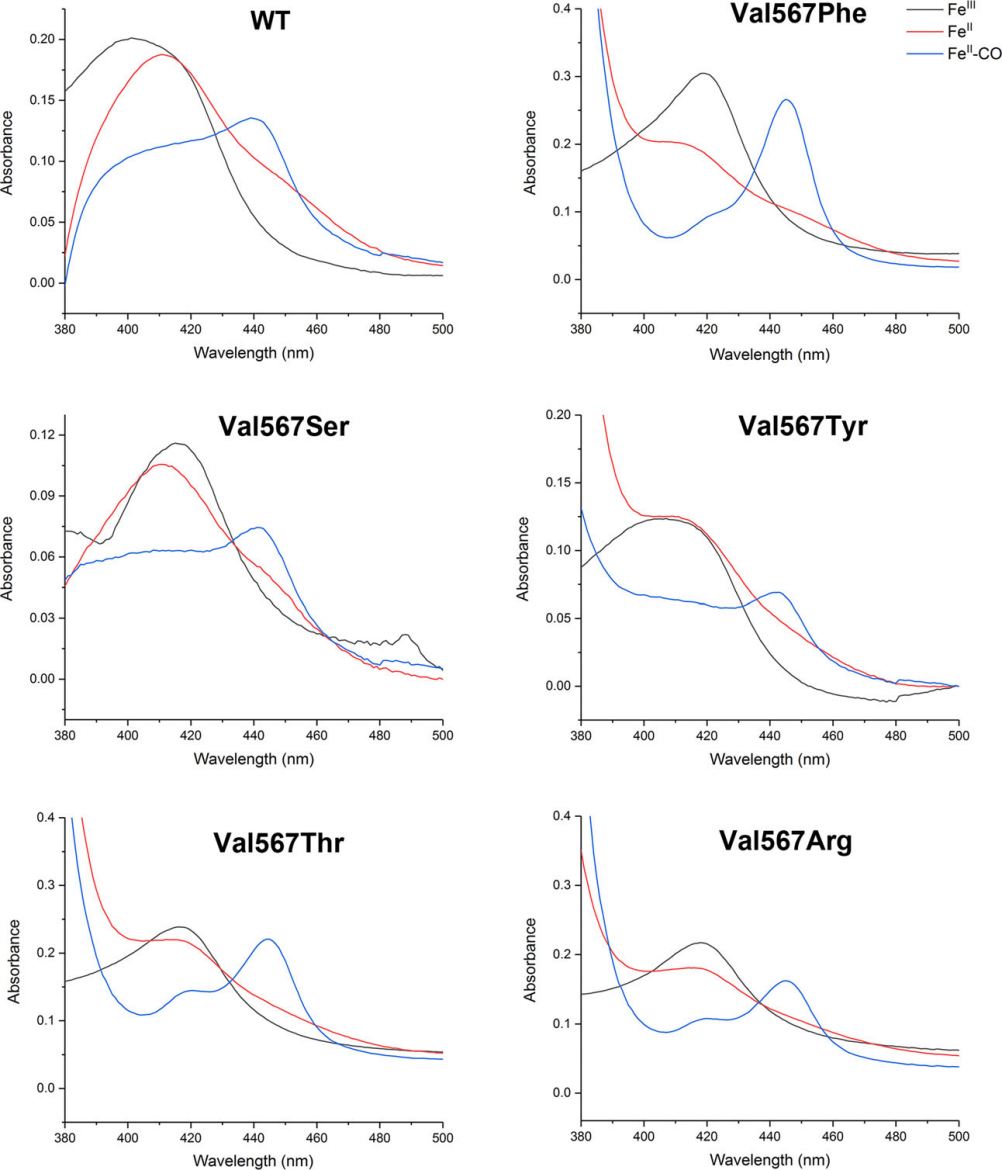
Optical absorbance spectra of purified WT nNOSoxy and its mutants Val567Ser, Val567Arg, Val567Tyr, Val567Thr, and Val567Phe. The black line shows the native Fe^III^ form, the red line the dithionite reduced Fe^II^, and the blue line the heme‐Fe^II^‐CO complexes. Spectra were recorded in 50 mm HEPES buffer pH 7.4 containing 0.1 m KCl and 10 μm BH_4_.

**Figure 5:**
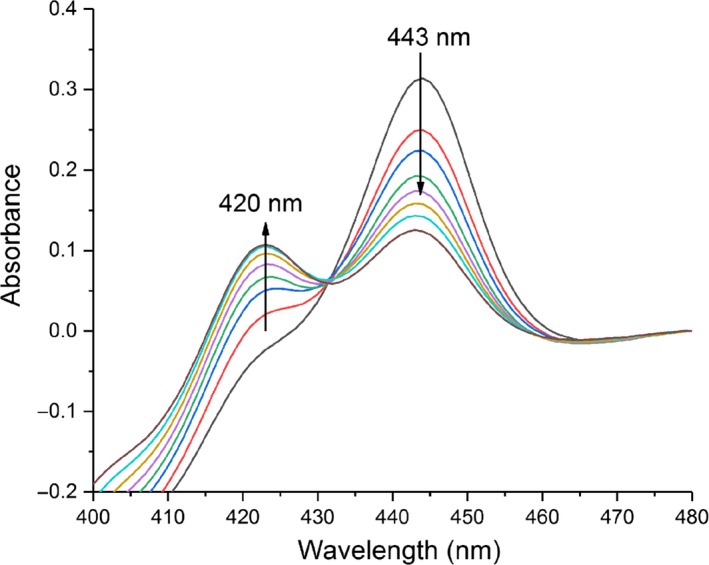
Time‐dependent disappearance of the heme‐Fe^II^‐CO complex of WT nNOSoxy. The black line shows the spectrum recorded just after addition of sodium dithionate and bubbling of CO in the sample cuvette containing WT nNOSoxy in 50 mm HEPES buffer pH 7.4 and 10 μm BH_4_. Spectra were then recorded every 10 min thereafter. The arrows show the disappearance of the 443 nm peak and appearance of the 420 nm peak. Data are representative of a typical experiment.

The optical absorption spectra of the purified heme‐Fe^III^ and Fe^II^‐CO complexes of WT nNOSoxy and its mutants were recorded (Fig. 4). WT nNOSoxy exhibited a broad Soret peak centered at 400 nm associated with a high‐spin heme‐Fe^III^ complex, whereas all the mutants absorbed at 415–419 nm, indicating that they predominantly existed as low‐spin heme‐Fe^III^ complexes. Addition of dithionite to the proteins caused heme reduction as evident by the shift in Soret peak to 414 nm, and addition of CO caused an immediate built‐up of the heme‐Fe^II^‐CO complexes with characteristic Soret peak at 443 nm (Fig. 4). However, this peak disappeared as a function of time with concomitant formation of an absorption peak at 420 nm in all proteins (Fig. 5). The presence of this 420 nm absorbing peak suggested the existence of another form of the NOS heme‐Fe^II^‐CO complexes for WT and mutants 61. To determine the impact of the Val567 mutations on the stability of Fe^II^‐CO complexes, we followed the time‐dependent disappearance of the 443 nm absorption peak. Previous studies have shown that the WT nNOS Fe^II^‐CO complex is destabilized in the absence of BH_4_ and l‐Arg 35, 61. Accordingly, we recorded the stability of the WT and mutants NOS heme‐Fe^II^‐CO complexes in the absence or presence of 10 mm l‐Arg (Table 1). As expected, in the absence of l‐Arg, the species absorbing at 443 nm of WT nNOSoxy and of the Val567 mutants were unstable with a shift of absorbance to 420 nm over time (Fig. 5) 61, 62. The heme‐Fe^II^‐CO complexes absorbing at 443 nm of the Val567Tyr and Phe mutants were less stable than those of the WT nNOSoxy, Val567Ser, Thr, and Arg mutants with the Val567Phe mutant being the least stable (Table 1). Interestingly, in the presence of l‐Arg, the stability of the heme‐Fe^II^‐CO complexes of WT nNOSoxy and Val567Ser and Thr mutants was strongly increased. By contrast, the stability of the heme‐Fe^II^‐CO complexes of Val567Phe was almost unchanged and that of Val567Tyr and Arg slightly reduced in the presence of l‐Arg (Table 1).

**Table 1:** Effects of mutations and l‐Arg addition on the stability of the heme‐Fe^II^‐CO complexes of WT nNOSoxy and its mutants

	− l‐Arg	+ l‐Arg
WT	45	95
Val567Ser	50	85
Val567Thr	35	70
Val567Phe	20	35
Val567Tyr	30	20
Val567Arg	40	25

Results are expressed as % of the absorbance of the peak at 443 nm remaining after 45 min at room temperature. WT nNOSoxy or its mutants in HEPES buffer pH 7.4 containing 10 μm BH_4_ alone or BH_4_ + 10 mm l‐Arg were equally distributed in sample and reference cuvettes. Both cuvettes were reduced with the addition of sodium dithionate, and CO was bubbled into the sample cuvette. Spectra were immediately recorded and every 10 min thereafter. Data are representative of a typical series of experiments.

### EPR spectroscopic properties of WT nNOSoxy and Val567 mutants

**Figure 6:**
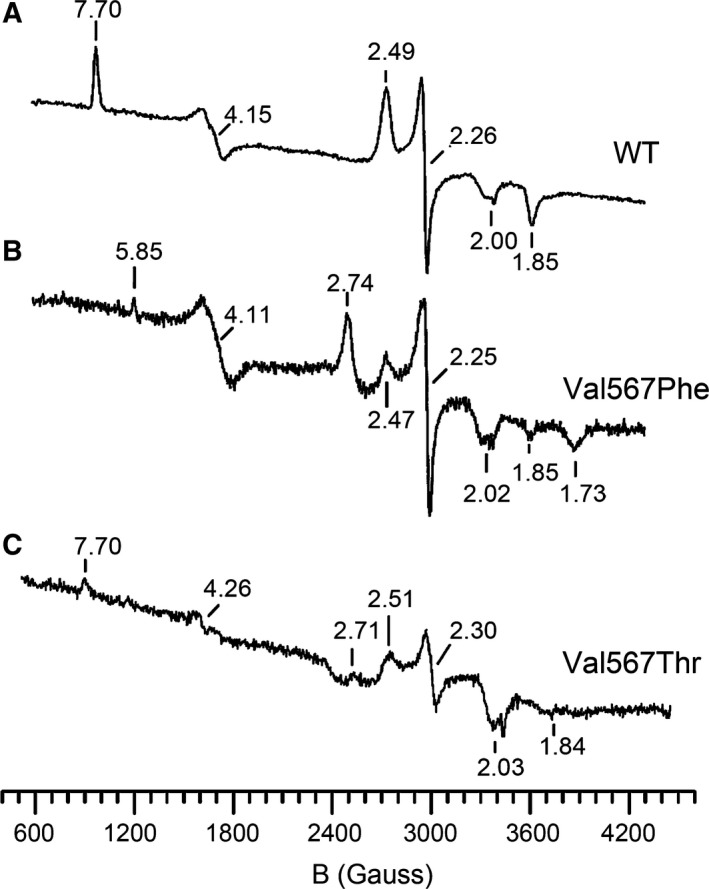
X‐band EPR spectra of native WT nNOSoxy (A) and its mutants Val567Phe (B) and Val567Thr (C). Spectra were recorded at 10 ± 2 K with 4 mW applied microwave power and in the presence of 10 μm BH_4_ cofactor. The numbers in panels A–C indicate *g*‐values.

The EPR resonance spectra of native WT nNOSoxy and of the different Val567 mutants were recorded at 10 K and in the presence of BH_4_ (Fig. 6). The oxidized form of WT nNOSoxy (Fig. 6A) showed signatures typical of ferric heme complexes in equilibrium between high (*S* = 5/2) and low (*S* = 1/2;) spin states 38. Both the high‐ and low‐spin components of the heme spin‐state equilibrium were visible and resolved. The resonances falling at *g* = 7.70, 4.15, 1.85 corresponded to the high‐spin fraction of the ferric heme, and the EPR signals with resonances at *g* = 2.49, 2.26, 1.85 corresponded to the low‐spin state fraction. By contrast, the EPR spectra of the Val567Phe mutant showed an EPR spectrum clearly distinct from that observed with WT nNOSoxy (Fig. 6B) with the characteristic high‐spin signal set around *g* = 7.70 not discernable. A weak resonance at *g* = 5.85 was observed, and a relatively strong and asymmetric EPR signal emerged at *g* = 4.11 which could correspond to adventitious rhombic high‐spin ferric iron attributed to contamination of nonspecifically bound iron. A cluster of poorly resolved signals appearing in the range 1.7 < *g* < 2.8 indicated the presence of several conformations for the low‐spin components, distinct of those observed for WT nNOSoxy. The Val567Thr (Fig. 6C) and Val567Ser mutants (data not shown) exhibited very weak high‐spin signatures falling at *g* = 7.70 and other heme high‐spin signals at *g* = 4.26 in addition to several signals observed in the region *g* = 2.8–1.8 corresponding to mixtures of several low‐spin heme species, distinct from those observed for WT nNOSoxy. Similar mixtures of low‐spin heme species were observed for the Val567Tyr and Arg mutants without any signal around *g* = 7.7 (data not shown).

### Affinity of WT nNOSoxy and Val567 mutants for imidazole

Determination of the ImH binding constants (*K*_s_ values) for WT and mutants was obtained by stepwise additions of ImH to the native ferric proteins. All the proteins formed a low‐spin heme‐Fe^III^‐ImH complex with a shift from 400 (WT) or 415 (mutants) nm to 425–430 nm characteristic of heme–thiolate proteins with nitrogenous ligands 37. WT nNOSoxy displayed *K*_s_ values close to 75 μm. The Val567Thr mutant displayed an almost identical affinity for ImH, whereas the Val567Ser, Val567Phe, and Val567Arg mutants displayed twofold to fourfold lower affinity. Interestingly, ImH displayed a much higher affinity for the Val567Tyr mutant (Table 2).

**Table 2:** Dissociation constants *K*_s_ (in μm) measured by UV‐visible difference spectroscopy following the formation of the heme‐Fe^III^‐ImH complexes by WT nNOSoxy and the studied mutants

	WT	Val567Ser	Val567Thr	Val567Tyr	Val567Phe	Val567Arg
*K*_s_	76.3 ± 5.4	140 ± 16	93.0 ± 24.7	11.1 ± 3.0	152 ± 26	262 ± 77

### Affinity of WT nNOSoxy and Val567 mutants for Guanidines 1–12

**Figure 7:**
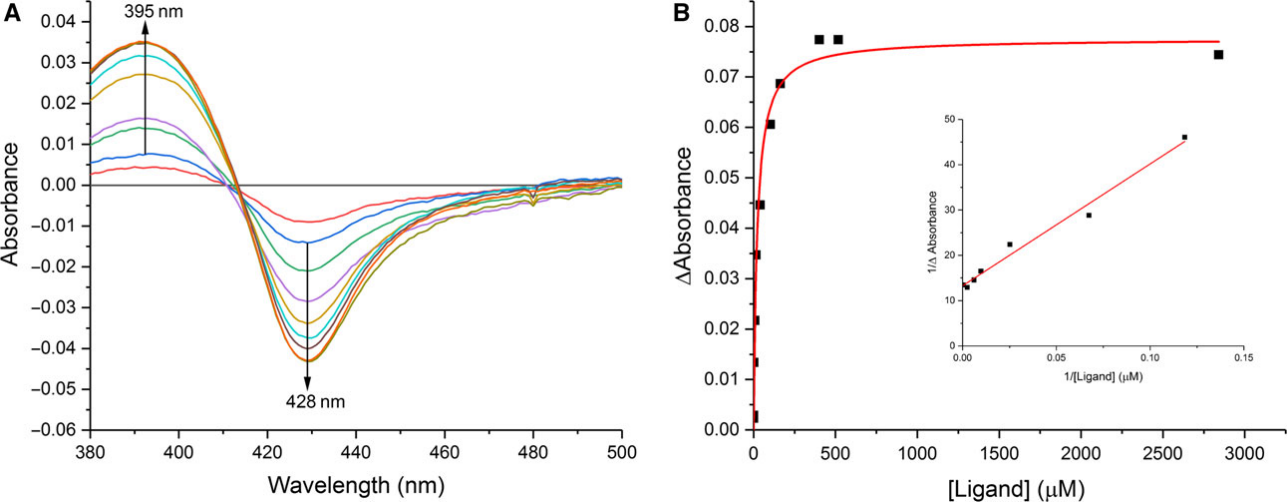
(A) UV‐visible difference spectra obtained upon addition of increasing concentrations of Cyclopropyl‐Guanidine **8** to 1 μm WT nNOSoxy containing 0.5 mm imidazole. (B) Plot of differences in absorbance (ΔAbsorbance) between 428 and 395 nm as a function of the concentration of **8**, and replot of 1/ΔAbsorbance versus 1/[ligand] giving a *K*_s,app_ value of 43.3 μm.

The apparent dissociation constants (*K*_s,app_) of l‐Arg **1** and its derivatives **2–12** for WT nNOSoxy and mutants were determined by difference spectroscopy after the addition of 0.5 mm ImH to completely convert their heme‐Fe^III^ complexes into low‐spin hexacoordinated heme‐Fe^III^‐ImH complexes as described previously 37. The stepwise addition of l‐Arg to the Fe^III^‐ImH complex of WT nNOSoxy and mutants gave rise to difference spectra characterized by a peak at 390–395 nm and a through at 420–427 nm 37, except Val567Arg which showed no discernible difference spectra. Similar titrations of WT nNOSoxy and mutants in the presence of ImH were performed with the substrate analogues **2–12**. A typical result is shown in Fig. 7A, which shows the difference spectra observed after stepwise additions of cyclopropyl‐guanidine **8** to WT nNOSoxy containing 0.5 mm ImH. The appearance of a peak at 395 nm and a through at 428 nm (Fig. 7A) indicated that the studied guanidine bound to the protein in close proximity to the heme and shifted the spin‐state equilibrium toward the high‐spin state with displacement of bound ImH 37. Plotting the absorption changes between peak and trough as a function of added guanidine could be fitted to a hyperbolic one‐site binding model (Fig. 7B) allowing the determination of the apparent dissociation constants (*K*_s,app_) values shown in Table 3.

**Table 3:** Apparent dissociation constants (*K*_s,app_) of substrate analogues **1–12**

Compounds	No	WT	Val567Ser	Val567Thr	Val567Tyr	Val567Phe	Val567Arg
**l** **‐Arginine**	**1**	1.7 ± 0.6	3.4 ± 1.2	3.6 ± 0.8	402 ± 58	145 ± 50	> 1 mm
**NOHA**	**2**	11.8 ± 2.4	5.8 ± 1.7	6.9 ± 2.5	214 ± 42	> 1 mm	1.2 ± 0.1
**N‐nitro arginine**	**3**	1.4 ± 0.5	0.42 ± 0.14	0.96 ± 0.33	9.7 ± 1.6	19.8 ± 3.4	1.9 ± 0.5
**N‐Methyl arginine**	**4**	0.57 ± 0.08	0.41 ± 0.06	0.13 ± 0.03	5.5 ± 1.9	48 ± 17	71 ± 25
**n‐Propyl‐guanidine**	**5**	317 ± 60	122 ± 8	267 ± 63	295 ± 16	>1 mm	326 ± 51
**n‐Butyl‐guanidine**	**6**	269 ± 20	322 ± 31	23.0 ± 5.6	>1 mm	>1 mm	>1 mm
**n‐Pentyl‐guanidine**	**7**	>1 mm	>1 mm	>1 mm	>1 mm	>1 mm	>1 mm
**Cyclopropyl‐guanidine**	**8**	43.3 ± 6.5	21.6 ± 3.9	20.2 ± 3.9	24.3 ± 1.4	6.4 ± 0.7	237 ± 85
**Isopropyl‐guanidine**	**9**	47 ± 12	34.2 ± 4.2	99 ± 19	147 ± 43	293 ± 34	> 500
**4‐trifluoromethyl‐phenyl‐guanidine**	**10**	>1 mm	>1 mm	>1 mm	n.d.	>1 mm	>1 mm
**4‐fluorophenyl‐guanidine**	**11**	29.0 ± 3.8	2.2 ± 0.4	n.d.	>1 mm	165 ± 87	n.d.
**4‐methoxyphenyl‐guanidine**	**12**	17.9 ± 5.9	7.8 ± 1.2	189 ± 63	n.d.	>1 mm	>1 mm

*K*_s,app_ (in μm) were measured by UV‐visible difference spectroscopy following displacement of the low‐spin heme‐Fe^III^‐ImH complex to the high‐spin heme‐Fe^III^ complex upon addition of substrate analogues **1–12**, as described in Materials and Methods. Error represents the error associated with the fit curve used to calculate the *K*_s,app_., and mean values of two to three experiments. n.d., not determined.

l‐Arg tightly bound WT nNOSoxy, and a threefold lower affinity was observed for the Val567Ser and Thr mutants. Very low affinities of l‐Arg for the Tyr and Phe mutants were measured, and no interaction could be observed between l‐Arg and the Val567Arg mutant. The substrate intermediate NOHA **2** displayed a lower affinity for WT nNOSoxy than l‐Arg but bound the Ser and Thr mutants with an almost identical affinity as that of l‐Arg. However, no binding could be observed between NOHA and the Phe and Arg mutants. The usual inhibitors N^ω^‐nitro‐l‐Arg **3** and N^ω^‐methyl‐l‐Arg **4** were potent ligands for WT nNOSoxy and the studied mutants with a threefold to 70‐fold higher affinity than l‐Arg for the Ser and Thr mutants (Table 3). The alkyl‐guanidines **5–9** that do not bear an α‐amino acid function displayed strongly decreased affinity for WT nNOSoxy and most of the mutants (60‐ to 160‐fold relative to l‐Arg). The affinity of simple alkyl‐guanidines **5–7** depended on the length of the alkyl chains with highest affinity obtained with n‐propyl‐guanidine **5**. Interestingly, isopropyl‐ and cyclopropyl‐guanidines **8** and **9** displayed good affinity for Val567Ser with cyclopropyl‐guanidine **9** tightly binding Phe and Tyr mutants. Introduction of the bulky aryl‐guanidines **11** and **12** 20‐fold decreased binding affinity for WT nNOSoxy in comparison with l‐Arg but led to an almost identical affinity as that of l‐Arg for the Ser mutant. However, these aryl‐guanidines **11** and **12** poorly bound the four other mutants while aryl‐guanidine **10** that bears a CF_3_‐group at the paraposition of the aryl ring did not interact with any of the studied proteins at the highest concentration tested (Table 3). The Val567Ser and Val567Thr mutants showed minimal deviation from WT nNOSoxy for binding of substrate analogues. By contrast, introduction of the aromatic amino acid residues Phe and Tyr led to strong deviations from WT and much more pronounced effects were observed when introducing the positively charged Arg.

### Modeling of the effects of Val567 mutations on the distal pocket

**Figure 8:**
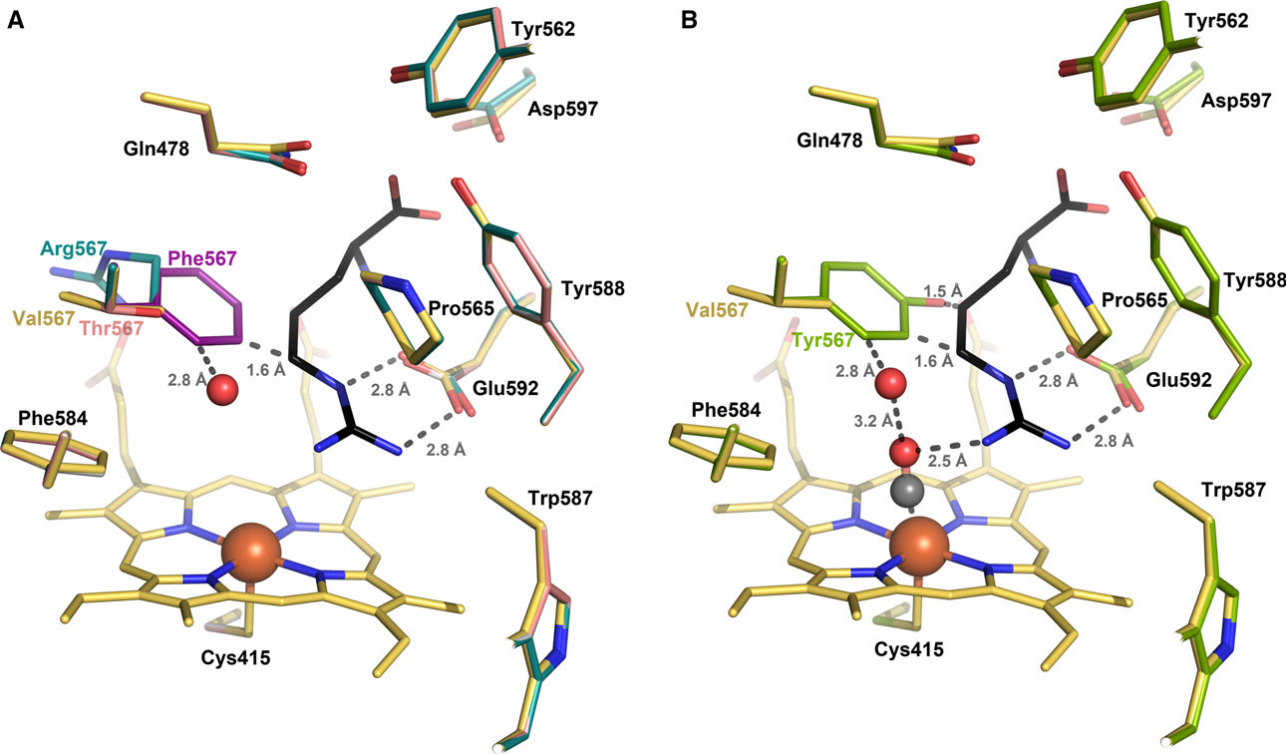
Structure of the active site of nNOSoxy (PDB id: http://www.rcsb.org/pdb/search/structidSearch.do?structureId=3HSO, yellow) and homology models of the mutants generated with SWISS‐MODEL without substrate. The l‐Arg has been added for comparison (black, PDB id: http://www.rcsb.org/pdb/search/structidSearch.do?structureId=1OM4). (A) Val567Thr (pink), Val567Arg (turquoise) and Val567Phe (purple). (B) Val567Tyr (green) and CO (gray, PDB id http://www.rcsb.org/pdb/search/structidSearch.do?structureId=3HSN). The weak bonds between the l‐Arg and the Glu592 and the CO‐molecule are shown as dashed lines, and the closest distance between the l‐Arg and the modeled Phe and Tyr mutants is indicated.

Modeling of the effects of Val567 mutations on the structure of distal heme pocket with SWISS‐MODEL showed that the Ser and Thr mutations did not lead to any significant differences from the Val residue except changing the polarity of the hydrophobic pocket (Fig. 8). The Arg mutant positioned its side chain away from the hydrophobic pocket and the substrate binding site (Fig. 8). The side chain of Phe and Tyr mutants on the other hand were positioned so that they would interfere with the l‐Arg binding site. A simple geometry optimization in the presence of l‐Arg showed that the position of both Phe and Tyr would move slightly to mutually fit in the heme pocket (data not shown).

## Discussion

Previous works have demonstrated that the hydrophobic isopropyl side chain of Val567 residue in the hydrophobic distal pocket of mNOSs plays a key role in recognition and transformation of substrates and stability of the Fe^III^‐NO complex 24, 49, 50, 51. Crystal structures also revealed a structural water molecule in this hydrophobic pocket that can modify the active‐site geometry and contribute to the binding affinity of ligands 63. In the present study, we have compared the spectroscopic and binding properties of five mutants of WT nNOSoxy that differ by the nature of this Val567 residue. WT nNOSoxy and its mutants were tested against a series of l‐Arg analogues that differ by modifications of the α‐amino acid function, the length of the side chain, and the presence of various substituents on the guanidine function (Fig. 3). Some of these compounds have previously been reported to have different binding modes to the NOS active site (Fig. 2B) 24, 64.

UV‐visible spectrum of WT nNOSoxy showed a broad Soret absorption peak around 400 nm, indicating that it existed as a mixture of high‐spin and low‐spin heme‐Fe^III^ complexes 37. Val567Ser, Thr, Phe, Tyr, and Arg mutants predominantly existed as low‐spin heme‐Fe^III^ complexes with broad absorption around 415 nm. The EPR study more precisely identified differences in the heme environment of the proteins. The heme‐Fe^III^ complex of native WT nNOS was predominantly in its high‐spin state. Under identical conditions, the studied Val567 mutants showed very weak high‐spin signatures with mixtures of several low‐spin species clearly indicating strongly altered heme environments.

After dithionite reduction and bubbling of CO, all the studied proteins formed the characteristic heme‐Fe^II^‐CO complex absorbing at 443 nm (Fig. 4) with almost identical stability (Table 1). However, differences were observed following the addition of l‐Arg. Addition of l‐Arg stabilized the heme‐Fe^II^‐CO complex of WT nNOSoxy and its Val567Ser and Thr mutants, whereas the addition of l‐Arg to the Val567Phe/Tyr and Arg mutants had almost no effect (Table 1). From the homology models (Fig. 8), the Tyr OH‐group of the Val567Tyr mutant could make a 2.6 Å H‐bond to one of the propionates of the heme group. In the presence of l‐Arg, this proposed orientation of the Tyr mutant would not be feasible if l‐Arg binds similarly to that observed in the WT. This is due to the close distance between the two residues (1.6 Å) (Fig. 8). A simple energy optimization showed that in the presence of l‐Arg, the Tyr aromatic side chain and the l‐Arg would move away from each other, thereby destabilizing the Tyr conformation and disrupting the H‐bond between l‐Arg and CO as present in the WT structure (data not shown). This might account for the lower stability of the heme‐Fe^II^‐CO complex of the Tyr mutant in the presence of l‐Arg (Table 1). In the case of the Val567Arg mutant, repulsion between the two positively charged guanidino moieties might also explain the lower stability observed for the heme‐Fe^II^‐CO complex of this mutant in the presence of l‐Arg.

The twelve studied guanidines (Fig. 3) displayed distinct WT and nNOSoxy mutant affinities. WT nNOS oxy and the Val567Ser/Thr/Tyr and Arg mutants bound l‐Arg or analogues bearing and α‐amino acid function (**1–4**) with affinity in the 0.1–10 μm range. The introduction of polar residues Ser and Thr resulted in a fourfold to fivefold decreased affinity for l‐Arg compared to WT (Table 3). This might be due to distortion of the H‐bond network including a water molecule close to l‐Arg by the OH‐group of Ser or Thr residues. Altering the water molecule(s) position might alter the hydrogen bonding from a moderate to a weaker hydrogen bond, resulting in reduced binding affinities. Introduction of a steric hindrance such as Phe and Tyr might also result in displacement of the conserved water molecule believed to aid in the correct positioning of the ligand in the active site (Fig. 8), thus resulting in the 100‐ to 300‐fold decrease in affinity. The decrease could also be explained by the close proximity of the aromatic amino acid to the substrate as seen from the homology modeling in Fig. 8, which shows that the aromatic side chain would need to reposition to allow for substrate binding as described above. A weakening of the binding of ImH, l‐Arg, and NOHA in the Val567Phe mutant was also observed for the full‐length nNOS in a previous study, indicating a similar effect of the mutation in the oxygenase domain alone and in full‐length nNOS (Table S1) 48. The origins of the differences observed in binding full‐length and the oxygenase domain may arise from differences in the preparation of proteins, or from the effects of the reductase domain on the conformation of the protein and/or access to the active site. However, the Val567Phe mutation slightly reduced the affinity of ImH (twofold), a ligand of the ferric heme, and more dramatically (100‐ to 300‐fold) those of l‐Arg and NOHA, two ligands of the distal cavity, highlighting the key role of Val567 in binding this cavity.

The decrease in binding affinity observed for the simple alkyl‐guanidines **5–9** could be explained by the active‐site geometry (Fig. 2A). When binding native substrates, l‐Arg and NOHA, crystallographic studies have identified three sites of interaction at the heme distal cavity: One is the binding site for the α‐amino acid moiety; the second is right above the heme plane where the guanidino group establishes three hydrogen bonds; and third is the distal hydrophobic pocket including a structural water molecule (Fig. 2A). Structural changes in the amino or guanidino end of a substrate have been shown to have distinct effects. While the guanidino end governs the precise positioning of the substrate over the heme, the amino acid end is needed to stabilize the H‐bonding interaction between the heme and the pterin cofactor 65. Upon complete deletion of the α‐amino acid function, the end of the propyl, butyl, and pentyl groups of **5–7** curls toward the Gln478 and Val567 away from the amino acid‐binding pocket 24 causing the Gln478 to swing slightly away avoiding close van der Waals contact with the ligand. The Val567 possibly provides favorable hydrophobic interactions based on the distance between WT protein and ligand (~ 3.7 Å for butyl) 24. The introduction of a Val567 mutation appears to have limited impact on these simple alkyl‐guanidines and the binding of their alkyl chain in the small hydrophobic pocket in close proximity to the heme (Fig. 2B). Previous studies of the binding of **8** and **9** revealed a novel binding mode where the terminal guanidino group NH_2_ and the NH both bind to the Glu592 (Fig. 2B) causing both CH_3_ groups of the isopropyl moiety of **9** and the CH_3_ and α‐NH of Val567 to be in close proximity to 3.52 Å and 3.69 Å, respectively 24. The novel binding mode causes a shift in the structural water molecule position (Fig. 2B, water W1b). The introduction of the aromatic residues Phe and Tyr are believed to, in turn, completely remove this structural water molecule. The increase in binding affinity observed for cyclopropyl‐guanidine **8** and the Phe and Tyr mutants is believed to be due to pi‐stacking.

## Conclusion

Our series of simple guanidines without an α‐amino acid function is a useful tool in comparing the changes in binding introduced by the Val567 mutations. The introduction of polar, aromatic, or charged residues results in changes in the distal H‐bond network of the heme distal cavity and in a displacement of a structural water molecule, thus accounting for the changes in binding affinity observed. This might also change the conformation of the heme porphyrin plane and introduce distortions with axial ligands, with consequences in the stability of the heme‐Fe^II^‐CO complexes. Although this study shows that several of the compounds have the ability to bind to the nNOS active site, further studies are needed to determine their validity as potential substrates as there is no clear relationship between the affinity of a compound and their ability to generate NO 44, 46, 48

In conclusion, all mutations of Val567 residue of nNOSoxy affect the electronic properties of the heme as shown by EPR and UV‐visible spectroscopy, the stability of the Fe^II^‐CO complexes and the binding affinities for different substrate analogues. Together, these data demonstrate the importance of the Val567 residue and of the hydrophobic pocket for binding and stability of ligands, including gaseous ligands such as CO, O_2,_ and NO, and thus NO formation.

## Author contributions

J‐LB, KKA, and IKO conceived and planned the experiments and IKO carried out the experiments while H‐PH carried out the structure modeling. IKO and J‐LB contributed to the sample preparations. IKO, H‐PH, J‐LB, and GZ contributed to the interpretation of the results. IKO wrote the manuscript with support of H‐PH, J‐LB, and GZ. All authors provided critical feedback and helped shape the research, analysis, and manuscript.

## Supporting information


**Table S1**. Spectral dissociation constants (*K*s) of ImH and apparent dissociation constants (*K*s,app) of l‐Arg and NOHA with full‐length WT nNOS, WT nNOSoxy, and with their corresponding Val567Phe mutants.
