# Supplementary material for: Importance of Val567 on heme environment and substrate recognition of neuronal nitric oxide synthase
Source: FEBS Open Bio. 2018 Aug 20;8(9):1553–66. doi: 10.1002/2211-5463.12503 (PMC6120233; doi:10.1002/2211-5463.12503)
Supplement: Supplementary file 1 — Table S1. Spectral dissociation constants (Ks) of ImH and apparent dissociation constants (Ks,app) of l‐Arg and NOHA with full‐length WT nNOS, WT nNOSoxy, and with their corresponding Val567Phe mutants. [file FEB4-8-1553-s001.pdf]

Supplementary information

Table S1: Spectral dissociation constants (K_s) of ImH and apparent dissociation constants ($K_{s,app}$) of L-Arg and NOHA with full-length WT nNOS, WT nNOSoxy, and with their corresponding Val567Phe mutants.

Proteins	ImH	L-Arg	NOHA	Reference
full-length WT nNOS	160 ± 50	3.5± 0.5	1.2 ± 0.3	48
WT nNOSoxy	76.3 ± 5.4	1.4 ± 0.4	11.8 ± 2.4	this work
full-length Val567Phe nNOS	480 ± 40	1400 ± 150	350 ± 50	48
Val567Phe nNOSoxy	152 ± 26	144.6 ± 50.2	> 1000	this work

Apparent dissociation constants ($K_{s,app}$ values, in μM) of L-Arg and NOHA with full-length nNOS and its Val567Phe mutant were measured using proteins (0.8–1 μM) previously shifted to the low-spin state by the addition of 0.5 mM (WT) or 1 mM (Val567Phe) ImH. Data are means ± SD from three experiments [48].
